# Assistive Robotics for Healthy Aging: A Foundational Phenomenological Co-Design Exercise

**DOI:** 10.2196/77179

**Published:** 2026-01-28

**Authors:** Stephen Potter, Mark Hawley, Angela Higgins, Farshid Amirabdollahian, Mauro Dragone, Alessandro Di Nuovo, Praminda Caleb-Solly

**Affiliations:** 1 Sheffield Centre for Health and Related Research University of Sheffield Sheffield United Kingdom; 2 School of Computer Science University of Nottingham Nottingham United Kingdom; 3 Robotics Research Group University of Hertfordshire Hatfield United Kingdom; 4 School of Engineering and Physical Sciences Heriot-Watt University Edinburgh United Kingdom; 5 School of Computing Sheffield Hallam University Sheffield United Kingdom

**Keywords:** robotics, assistive technology, frail older adults, activities of daily living, independent living, human centered design, co-design, lived experience

## Abstract

**Background:**

Assistive robotics for helping older people live well and stay independent has, to date, failed to fulfill its promise: there are few assistive robots in everyday use. In part, this failing can be attributed to inadequate or missing co-design activities that would ensure that these technologies and any services that incorporate them are developed with prospective end users, addressing their actual needs and wants, and not merely for them, and based on lazy assumptions about heterogeneous user groups.

**Objective:**

This exercise aimed to address some of these limitations by taking a “phenomenological snapshot” of what it means to be an older person in the current sociotechnological context, and making this snapshot, along with the co-design materials developed, available to the wider assistive robotics community to provide solid foundational evidence for steering the development of assistive robotics in more productive directions.

**Methods:**

Two rounds of co-design workshops have been conducted with older people and their caregivers, based on an innovative methodology that used personas and speculative designs to explore sensitive everyday difficulties faced by participants and highlight some of their general wishes for and concerns about assistive robotics. The data collected during the workshops were analyzed, and key themes were extracted.

**Results:**

Analysis of the workshop data gives access to the lived experience of older people and their caregivers, and their opinions about domestic robotics and assistive technologies more generally. The findings are organized thematically as *everyday difficulties*, the daily problems faced by older people; *ideas for aging better*, older people’s own suggestions for how their lives could be improved; and *living with technology*, their preferences and requirements for assistive robots, along with their concerns about what the introduction of robots might mean, both for themselves and for society more widely.

**Conclusions:**

We believe that our findings provide solid foundational evidence for the development of assistive robotics for older people. We are in the process of disseminating these results through various channels to the wider assistive robotics community; ultimately, the success of our activities will be demonstrated only through the development of acceptable, useful, and viable assistive robotics for older people.

## Introduction

### Emergence Network

Emergence is a UK-wide initiative created to foster innovation in the field of assistive robotics for older people, especially people living with frailty, and to facilitate the “emergence” of robots from research laboratories into service. The network is coordinated by a team of academic researchers and, in addition to other academic partners, members include health, social care, and housing providers, regulators, and robotics industry representatives, who provide clinical focus and business acumen to anchor its activities. The network was launched in early 2022 and is led by the University of Nottingham, with the support of coinvestigators from Heriot-Watt University, the University of Hertfordshire, the University of Sheffield, and Sheffield Hallam University.

More specifically, the objectives of Emergence are to promote the development of domestic assistive robotics that will improve the quality of life and independence of older people by enabling better self-management of frailty and age-related issues, by helping with activities of daily living, by assisting rehabilitation activities, and by supporting health care professionals to make tailored interventions to slow decline or to improve resilience. The primary impetus behind the establishment of the network was the observation that, despite the undoubted effort and resources that had been directed at the task, few assistive robots for older people are in general use (see, for example, the book by Wright [1]). Plausible factors that may have contributed to this include a poor appreciation of the needs and wants of older people, of the wider psychological, social, and clinical considerations, and of the challenges and practicalities of operation in noisy real-world domestic environments.

One fundamental pillar of the Emergence network is the proposition that this general lack of understanding of use contexts can be addressed through a rigorous and ongoing co-design exercise involving people from the target user groups, with the results of this exercise guiding assistive robotics development.

### Frailty

Frailty is “a common clinical syndrome in older adults that carries an increased risk for poor health outcomes, including falls, incident disability, hospitalization, and mortality” [2]. It is characterized by the “loss of biological reserves across multiple organ systems,” leading to clinical vulnerability to stressful episodes [3]. While it is not an inevitable consequence of aging, frailty is common among older people, and its extent and severity can vary significantly from individual to individual. Many older people who live with a health condition (or with multimorbidity) or with a disability will also be living with frailty: the one can contribute to or exacerbate the other. It can affect a person’s physical and cognitive abilities, to the detriment of their wider health and well-being, and can limit their day-to-day functioning [4]. Frailty, when it does occur, is not a static condition: it can become worse, but there is evidence to suggest that, with the right interventions, it can be reversed to some extent [3].

When it comes to characterizing frailty, there are currently 2 dominant models [5]. The “(cumulative) deficit model” of frailty [6,7] assumes that frailty derives from an accumulation of “deficits” (which might be disabilities, health conditions, or psychosocial factors) that, individually and cumulatively, increase a person’s vulnerability to stressful events. The second prevailing model is the “phenotype model” of frailty, in which an individual who manifests at least 3 of a set of 5 criteria for frailty (weight loss, low physical activity, exhaustion, slow walking speed, and physical weakness) is considered to be frail [4].

These models have their uses when considering whether an individual, in a health or care context, might require additional support or could benefit from some remedial intervention. However, they are less useful when it comes to understanding people’s lived experience of frailty—and, by extension, for developing practical assistive technologies, including robotics, to support people living with the condition. The impact of deficits or of physical manifestations will vary from person to person, depending on their living circumstances, their tastes, their responsibilities and roles, and their hobbies and interests. Hence, while both the deficit model and the phenotype model can provide suggestions of where assistance might be needed, they also promote a narrow (and negative) view of people with frailty as deficient, a view that excludes a more rounded consideration of their lives that acknowledges their abilities (see the study by Lang et al [8]).

To take an example, clinical tests might reveal a loss of grip strength. The everyday impact of this on an individual might be felt in their inability to open jars of their favorite food (which might then have the knock-on effect of reducing the amount they are eating, leading to weight loss). Whereas the clinical model might suggest more-or-less generic remedial strength exercises—that is, focusing on the deficit or symptom and “treating” it—a more complete understanding of the individual’s experience opens up the design space to suggest other, perhaps complementary, approaches encompassing the technological (a jar-opening robot), the environmental (redesigned food packaging), the social (a personalized meal delivery service), and so on. Any of these “interventions,” or perhaps some combination of them, might be more acceptable to, appropriate—and, ultimately, effective—for the individual in question. The goal of the research reported in this paper is to provide a co-design counterbalance to the prevalent clinical models in the form of a snapshot of the lived experience of older people, one that encompasses their views of the digital world and, especially, robotics.

### Assistive Robotics for Healthy Aging

Assistive robotics could help older people living with frailty, long-term health conditions, and disabilities to live more independent, more dignified, and more fulfilling lives. Moreover, services incorporating assistive robotics could play a role in plugging the gap in care provision that is being felt in many countries as their populations age, and they experience shortages of care staff. Many of the assistive robotics development projects undertaken have addressed one or other aspect of care provision for older people. These have typically taken the form either of, on the one hand, physically assistive robots (eg, for mobility support [9,10], exercise training [11] and help with eating [12]) or, on the other hand, of socially (or clinically) assistive robots—recent contributions have focused on stimulation [13], companionship [14,15], assessment tasks [16,17], monitoring [18-21], and remote visits or consultations [22]. It will be noted that both physically and socially assistive robots each tend to address only 1 or 2 aspects of frailty (care); as such, none of these robots can be said to be “for frailty” in its widest sense, if, indeed, such a thing is even possible. Notwithstanding these and other encouraging feasibility studies, undertaken both in laboratories and, on occasion, in people’s homes, care homes, or clinical settings, this effort has yet to translate into anything approaching scale-up of production and widespread adoption. Despite years of research and development—and not inconsiderable financial investment—few assistive robots are actually deployed in the real-world to provide care or assistance to older adults [1].

At a technical level, it is undoubtedly extremely challenging to provide robotic assistance in a safe, sensitive, timely, responsive, respectful, reliable, and trustworthy manner to, with, and around ordinary people—who may have sensory, cognitive, or physical impairments—as they go about their everyday lives. However, the task has not been made any easier by basing design decisions on often unfounded or simplifying preconceptions of older people’s needs, wants, and circumstances. In Emergence, we want to address this issue through the effective co-design of assistive robotics.

### Co-Design of Assistive Robotics

Conventional design practices can result in “solutions” that leave end users with a sense of alienation and exclusion (especially where new or unfamiliar technologies are deployed), and the (entirely accurate) impression that the product or service has been designed “for them” and not “with them.” It is likely that such products or services will not be adopted or quickly fall into disuse, unless their users have no alternative, when they will be used under duress and with little pleasure, and possibly with limited success. This failing becomes particularly significant in assistive technology contexts (and in health and care contexts more generally), where outcomes relate directly to the health and well-being of the end user. Co-design practices seek to address this failing by increasing the involvement of external stakeholders, and, in particular, end user representatives, in design and development processes, particularly during the earliest phases of design when, as is widely acknowledged, any incorrect decisions taken are significantly more difficult (and costly) to rectify later on. Co-design is a term given to a broad range of participatory techniques and methodologies whose underlying objective is to improve the acceptability and value of products and services by involving representative target users in their development [23]. During co-design, the role of the external actors can range from the relatively passive (such as critiquing alternative designs put to them) through more engaged modes (helping to define requirements or brainstorming potential solutions) to effectively becoming embedded in the design team and contributing to all stages of the development process.

There have been previous co-design exercises involving older people (for examples see other studies [22-27]). However, these exercises also underscore that co-design is not easy, with no single methodology; that access to the right stakeholders at the right time is not a given; that it is costly, in terms of both money and time; that participants find it difficult to shake preconceptions about robots and the roles they can play; and that, in the final analysis, it does not guarantee success.

### Aim

Given what is at stake, however, none of these is a reason not to do co-design; rather, they are reasons to devote sufficient resources and care to try to do it better. The work reported in this paper aims to provide assistive robotics for older people with sturdy, co-designed foundations. Specifically, we have performed an extensive early-stage co-design activity with older people through which we hope to understand some of the current everyday problems facing people and their general requirements and attitudes toward hypothetical robotics solutions to some of those problems. We have undertaken this activity with the intention of collating the results and then sharing them as widely as possible among the assistive robotics community and, in this way, to provide a rich seam of foundational evidence for researchers and developers who otherwise lack the skills, wherewithal, or opportunities for engaging in early-stage co-design with older people. Ultimately, we intend to steer assistive robotics for older people in directions that are more likely to lead to the development of assistive robot services that provide genuine value for their users.

## Methods

### Emergence Co-Design Exercise

The objective of the co-design exercise was to take a “phenomenological snapshot” of frailty and aging, that is, one that positions the individual at the center of the enquiry [28] to complement the clinical models described above by capturing people’s everyday experiences of frailty and aging. Moreover, the aim was to do this in such a way as to avoid some of the weaknesses of previous co-design in this field by expressly not focusing on any specific task or assuming as a basis any specific robot platform. This snapshot would encompass the physical, psychological, and social effects of aging, as well as the role that innovations (not necessarily technological) could play in mitigating negative effects and improving people’s lives, and any constraints under which such innovations must operate if they are to be acceptable. As stated, the primary purpose of capturing such a snapshot is to provide guidance and support for the early stage (conceptual design) development of assistive technologies, specifically assistive robotics. However, the snapshot could play a role beyond this, informing a variety of interventions and improvements, and educating those involved in the care and support of older people.

Moreover, it was imperative that the snapshot be accessible and communicable to a wide network of robotics and technology researchers, designers, and developers, and in such ways as to encourage the development of appropriate assistive robotics solutions. This, we hoped, would help to overcome the problems of a lack of expertise, experience, and resources for performing co-design, and of limited access to participants.

As mentioned above, there is no single methodology for co-design, although a number of different approaches have been suggested. Here, we adopted a selection of approaches and tools, intended to give us access to complementary information and in such a way as to hold the participants’ interest and keep them engaged.

### Co-Design Methodology

#### Overview

Workshops were run in 3 different locations (corresponding to the locations of academic partners in the Emergence network) in the United Kingdom. The workshops were “paired” at each location: an initial, “lived-experience” workshop was followed by a “speculative-critique” workshop.

#### Lived-Experience Workshops

The lived-experience workshops were designed to enquire into the needs and aspirations of older people living with frailty, the everyday realities of their lives, their living environments and social activities, and the benefits and frustrations of modern life, and, inevitably, of modern technologies (although the workshops were not designed to discuss technologies specifically). Participants were encouraged to identify any opportunities for assistive technologies, including robotics, to play a role in older people’s lives.

To facilitate these workshops, we used personas as a starting point for group discussions. Personas are descriptions of fictitious individuals and have been widely used to motivate or contextualize co-design or innovation processes [29]. A total of 10 personas were developed for the Emergence workshops. We decided to develop these by drawing on our previous research [30] and existing data-based resources such as the CURE-Elderly-Personas set [31], which were adapted and extended to be more relevant to the United Kingdom context and to focus on the specific characteristics of the populations of interest to Emergence.

**Figure 1:**
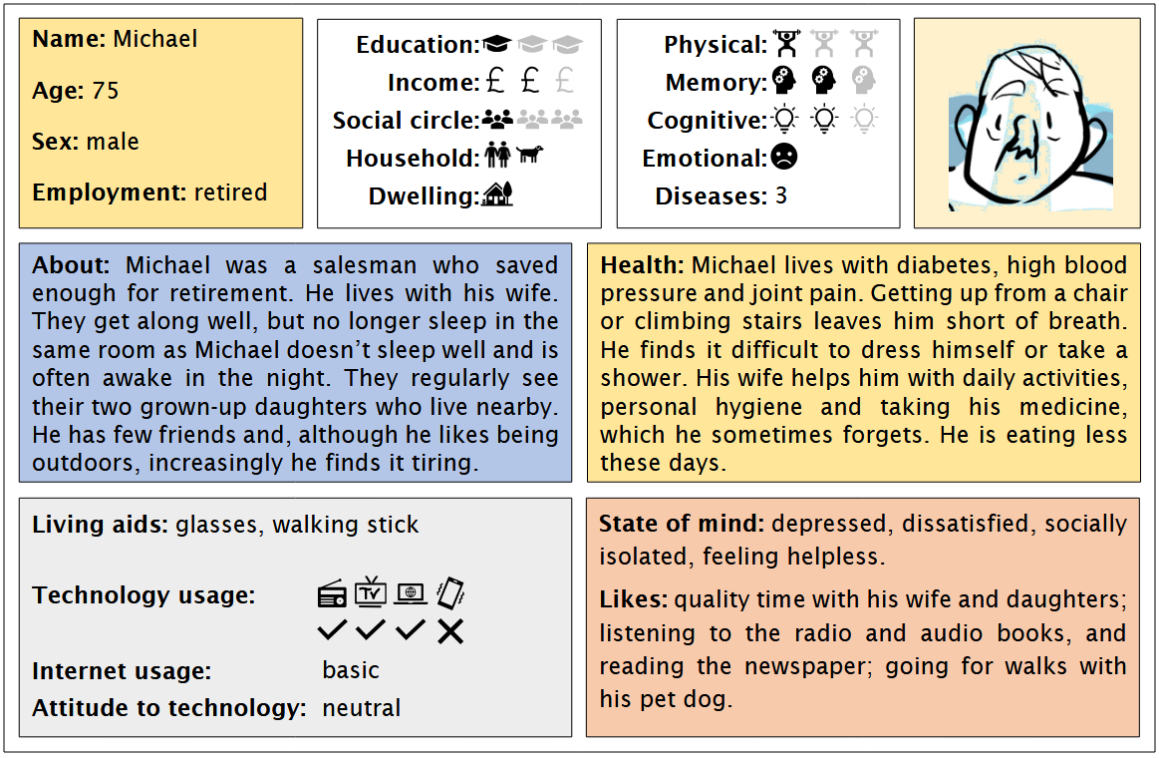
“Michael,” one of the personas developed for the lived-experience workshops.

**Figure 2:**
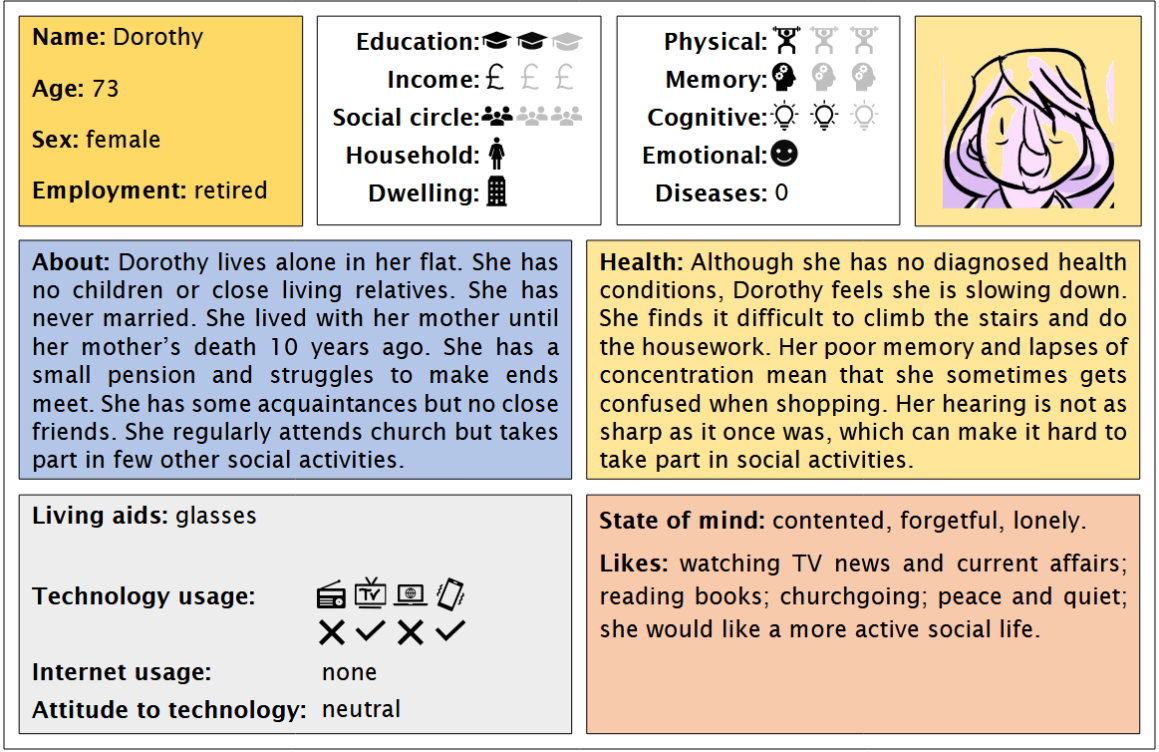
“Dorothy,” one of the personas developed for the lived-experience workshops.

These personas depict, in easy-to-digest formats, the backgrounds, health status, and characteristics of fictitious older people, each of whom can be classed into 1 of 3 broad categories: prefrail but managing; vulnerable or living with mild frailty; or living with moderate-to-severe frailty. It was envisaged that having personas with different experiences of frailty would allow us to explore different intervention contexts and make comparative assessments of needs and wants. Before use, the personas were validated to be representative of people living with frailty by the Emergence steering groups, comprising health and social care professionals and people with lived experience of frailty. The group also reviewed the terminology used for possible infelicities or offence. Examples of the personas developed for and used in the workshops are shown in Figures 1 and 2. The personas have been made publicly available to allow their use in other co-design exercises.

Personas can be used in different ways within design and innovation processes. Here, the primary role of the personas was to aid our participants within the co-design activities to “transfer” their own needs, wants, opinions, difficulties, and coping strategies onto fictional others, thereby avoiding potentially sensitive, embarrassing, or otherwise inhibiting discussions of a personal nature, allowing us obliquely to broach frailty and other issues related to aging. More specifically, they would be used in the context of discussions of different “episodes” of the persona’s typical daily routine:

- Getting up: including waking, toileting, washing and bathing, dressing, and medication.
- Mealtimes and snacks: including planning meals, food preparation, ordering food, utensil operation, cooking, eating, drinking, washing up, remembering to eat and drink enough, and maintaining a balanced diet.
- Household chores: including cleaning the house, heating the house, laundry, everyday repairs and maintenance, looking after pets, and household management (payment of bills, etc).
- Out and about: including getting around (walking, driving, and public transport), going to the hairdressers, going to the supermarket, going to the bank or post office, going to the doctor’s, buying items, carrying things, and outdoor exercise.
- Socializing and pastimes: including home entertainment (television, music, internet, and puzzles), gardening, meeting friends and family, receiving guests, indoor exercise, and outside entertainment (cinema, concerts, bingo, book groups, walking groups, etc).
- Bedtime: including taking medicine, switching off and locking up, climbing stairs, getting to sleep, and getting up in the night.

Working in small groups consisting of 3 or 4 participants, each of which would be allocated 2 personas and 3 daily episodes, the lived-experience workshop participants were asked to consider what difficulties the episodes might present for those personas and what opportunities they can see for making the personas’ lives easier. Following a break, the groups were then asked to consider each of the difficulties or opportunities and reach some consensus about: how frequently it occurs (daily, weekly, monthly...?); how prevalent it is (encountered by most, some, or just a few people?); and how consequential it is (does it have a large, medium, or small impact on people’s ability to get by?). The answers to these questions would allow us to assess the significance of the difficulties and opportunities, and hence to prioritize them as objectives for assistive technologies.

The group discussions were facilitated by members of the research team who took notes as participants spoke, displaying these on whiteboards for immediate validation by participants. The discussions were audio-recorded for future verification and analysis.

In addition, the workshops were documented by a professional illustrator with experience in supporting academic workshops. Graphic facilitation can capture ideas in the form of easily digestible pictures, and can visualize the progression of and relationships between concepts discussed [32]. These illustrations would also represent the findings of a workshop in an easily accessible format, for reflection (and validation) during and after the workshop [33]. This was felt to be a particularly important aspect in this case, since one of our objectives was to communicate, in as readily accessible a manner as possible, the results to the wider assistive robotics development community. The illustrator was briefed as to the purpose and structure of the workshops, and then given free rein to move among the group discussions and capture any parts of the discussions that seemed to them both significant and susceptible to pictorial expression (they would also incorporate text, often quoting participants verbatim, into their illustrations). The illustrator attended and illustrated all the lived-experience workshops; they were unable to attend the subsequent speculative-critique workshops (but would supply the illustrations used to facilitate these workshops, as described below).

#### Speculative-Critique Workshops

The speculative-critique workshops would be structured around several “speculative designs” of assistive robots proposed to address the problems or grasp the opportunities identified by participants in the initial lived experience workshops.

These designs were in the form of a brief textual description and a sketch of the robot in action, helping to convey both the appearance of the robot and its use and operation. These were not intended to constitute designs for robots that would necessarily be developed; indeed, although the robots were intended to be realistic, in the sense of their functioning and behaviors being more-or-less feasible in the short term given current developments and directions in robotics research and development, they did not need to be feasible at the time of the workshops given the current state-of-the-art. Instead, they were to serve as “provocations” (elsewhere, such design provocations in the context of participatory design have been termed “provotypes” or “provocative (proto)types” [34]). As such, these workshops would constitute “speculative (co-)design” activities. Speculative design [35] is an activity during which a design proposal is presented not as a candidate for subsequent product development but as a means to elicit concerns or highlight issues that might otherwise remain latent, but which must be acknowledged if the design process is to be successful. This approach was felt to be particularly appropriate for a field such as robotics, about which many people possess preconceived notions. Moreover, discussing potential applications of any new technology without concrete examples is difficult: the speculative designs would constitute a basis for grounding discussions around assistive robotics in something approaching reality. Attempts to ground co-design discussions around (prototype applications of) real, existing robots are common; however, the obvious limitation of this approach is that the robots in question will have already been developed to some degree and, even when they have been developed with assistive applications in mind, this is likely to have involved certain assumptions on the part of the designers. Moreover, they will almost certainly have technical shortcomings. Consequently, it is difficult to move the discussion beyond the specific limitations of that particular robot or envisaged application so as to capture more general requirements for assistive robotics. We believe that the speculative design approach gives participants more freedom to move beyond the particular and express more general opinions about future applications of technology [36].

The development of the speculative designs required a rapid analysis by members of the research team (SP and MH) of the content of the lived-experience workshops to identify candidate domestic tasks with which assistive robotics might help; a total of 6 such “robots” were identified:

- Motibot (“the motivational well-being and exercise robot”)
- Foodee (“your personal cooking assistant and dietary advisor”)
- EasyUp (a mobility assistance robot “for all life’s ups and downs”)
- AutoReach (a cleaning robot to “keep those hard-to-reach places spotless”)
- RoPet (a robot pet that is “your new faithful friend”)
- Toilittle (a discreet toileting robot that’s “there when most you need it”)

**Figure 3:**
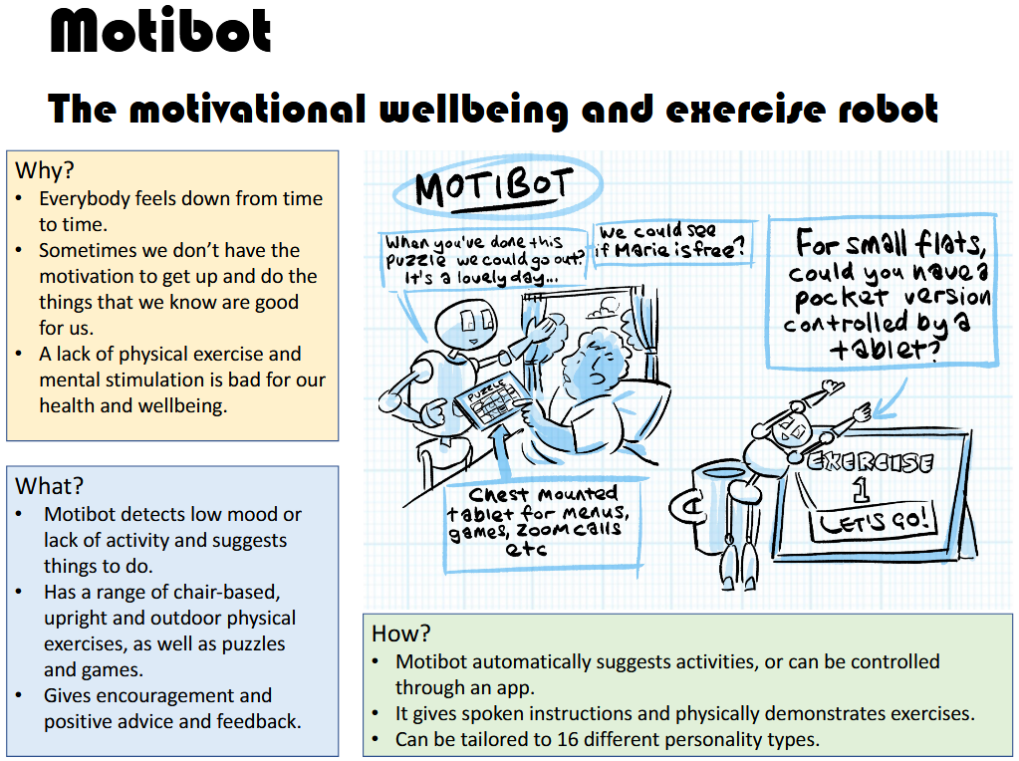
Motibot (“the motivational well-being and exercise robot”): one of the speculative designs developed for critique.

**Figure 4:**
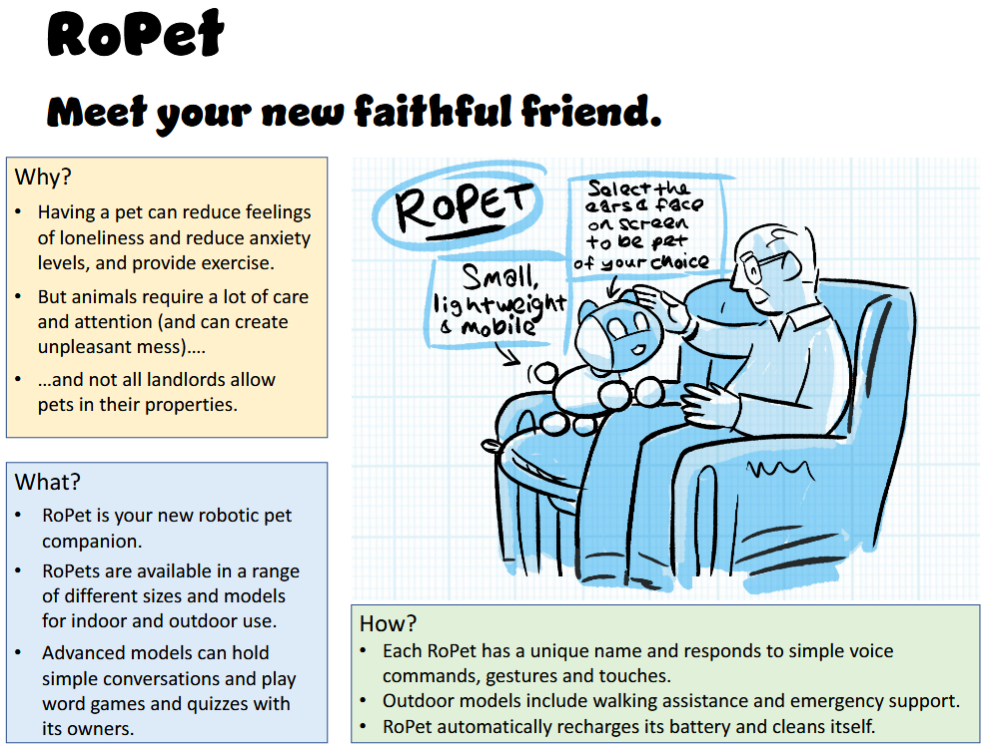
RoPet (“meet your new faithful friend”): one of the speculative designs developed for critique.

Each of these was summarized in words in terms of the problem or opportunity that the robot sets out to address (the “why” of the robot), its projected functionalities (the “what”), and how the robot would operate or be operated (the “how”). To aid in communicating the designs to workshop participants, these textual descriptions then became the brief for the illustrator to work up a sketch of the robot “in action,” working with the research team to imagine what form the robot could take. In undertaking the brief, across the 6 applications, the illustrator was asked to draw robots with a range of embodiments, sizes, interfaces, and movement styles to elicit responses to different design options. Figures 3 and 4 give examples of 2 of these speculative designs in the format in which they were presented at the workshops.

For the speculative-critique workshops, the participants were, once again, allocated to small groups, and asked to consider each of the speculative designs in turn, it first having been emphasized that the designs portray imagined, rather than real, robots. The participants were asked whether they would like to have the robot, and then asked for the reasons for their decision. Participants were encouraged to ask for additional information if this would help; facilitators were encouraged to extemporize answers. Using different-colored notepads, the facilitators noted separately positive and negative arguments, as well as quoting verbatim the words of participants where these seemed particularly pertinent. If at least 1 member of the group said they would accept the robot, the group was then asked about the following features of the robot:

- Appearance: what was liked and disliked about it, what size and color it should be, what materials it should be made of, and so on.
- Control: the extent and nature of the control that the end user and their caregivers should have over the robot, appropriate modalities for controlling the robot (voice, touchscreen, smartphone app, etc), and so on.
- Performance: its operating speed, acceptable noise levels, weight, communication, quality of movement, behavior both when in use and when not in use, and so on.
- Practicalities: how it would fit into people’s lives and homes, whether people would be comfortable operating and maintaining the robot, the sort of training and assistance they felt they might need, and so on.
- Concerns: any safety or security issues, whether the robot seemed trustworthy, who would be trusted to develop, supply, run, maintain the robot, and so on.

Once again, facilitators would note both positive and negative comments: the former would constitute design requirements (“should-haves” or “nice-to-haves”) while the latter constitute design constraints (“should-not-haves”).

#### Participants

Older people do not always recognize themselves as living with frailty and can resist being labelled as “frail”: the term has certain negative connotations, and not all people clinically classified as such would recognize (or welcome) the attribution [37]. Moreover, its severity can vary dramatically, from those who are moribund and almost completely dependent on others to those whose coping strategies are so successful that others–—and maybe even they themselves—might not consider them to be living with frailty. For these reasons, we avoided using the term “frailty” when engaging with older people and their caregivers in these co-design activities. Moreover, we chose not to recruit through health services because we would then be accessing only those who, for one reason or another, had come to the attention of those services and received an “official” diagnosis. Instead, we focused on recruiting through independent-living housing providers managed by Emergence partners and located conveniently close to the Emergence university research centers. People aged 55 years and older were considered “older people,” adopting the threshold age for residency used by one of the housing providers. Even if not living with frailty themselves, most people living in these contexts would almost certainly know of people among their friends and neighbors who are living with frailty. In addition, relatives and friends who play an active role in supporting older people, along with other caregivers, were invited to participate, especially where they could support older people to participate by helping with transport and mobility.

Participants were asked to attend both the lived-experience and the subsequent speculative-critique workshops at their locations. However, for various reasons, some participants were unable to attend both workshops. As far as possible, the workshops took place in locations that were convenient for participants to attend and which, as far as possible, were close to their “natural environments” (such as communal lounges or university collaborative spaces) and so conducive to eliciting their experiences, while also being appropriate for group discussions. Likewise, workshops were scheduled for times considered most convenient for participants. Each workshop typically lasted around 4 hours, including breaks and refreshments. There was an interval of approximately 2 weeks between the lived-experience workshop and the corresponding speculative-critique workshop, except for the last pair of workshops, which, due to scheduling pressures, were held as morning and afternoon sessions on the same day. Before the workshop, participants were asked to complete a consent form and a demographics questionnaire. At the end of the workshop, they were invited to give feedback about the nature, structure, and conduct of the workshop, and were each given a shopping voucher as an honorarium to acknowledge their contribution.

#### Data Analysis Method

The audio recordings of the workshops were transcribed by professional transcribers; the transcriptions, alongside the notes collected during the workshops, were then subjected to a thematic analysis by two of the research team, one with experience of assistive technology development (SP), the other with a background in industrial design and robotics (AH). The analysis broadly followed the Framework Method [38] as elaborated by Gale et al [39]. A phenomenological approach was adopted for analysis, with the analysts bracketing their own experiences and conceptions to reduce bias and focus on the content of the participants’ contributions, paying particular attention to their experiences and the meanings that they attached to these. Specifically, we adopted an interpretive phenomenological analysis, foregrounding the participants’ experiences and perceptions, while recognizing that the researchers play an active role in interpreting these [40,41]. This analysis was both deductive and inductive. Given the aims of the exercise, we had a particular deductive focus on the lived experience of aging and how this relates to opportunities for and constraints upon assistive robotics, as dictated by the structure of the workshops. However, within these broad topics, we were open to themes that were not explicitly foreseen as topics for discussion, and which arose naturally during the workshops. Codes were first inductively identified from the participants’ utterances and then checked against the notes taken by the facilitators. These were then clustered deductively into higher categories which broadly mirror the structure of the workshops, namely “difficulties with activities of daily life,” “opportunities for facilitating activities of daily life,” “assistive robotics design requirements,” and “assistive robotics design constraints.” The analysts worked independently at first, then collaborated to merge codes, resolving any discrepancies through discussion. Several iterations of analysis, during which codes and categories were amended, were performed until a reasonably stable analytical framework emerged, with the first 2 themes emended to “everyday difficulties” and “ideas for aging better,” respectively, and the last 2 merged into the more general “living with technology” category. The transcription and analysis of the data were carried out using the Lumivero NVivo qualitative analysis software package. Although the notes taken were displayed to participants for “real-time” validation during the workshops, it was unfortunately not feasible to ensure dependability by reconvening the participants and presenting the analysis back to them for their approval or correction of the framework.

### Ethical Considerations

This study was approved by the Ethics Committee of the University of Nottingham (CS‑2021‑R40). All participants provided written informed consent. Privacy and confidentiality were ensured by collecting no personally identifying data. Participation in this study was voluntary; each participant was given a shopping voucher (worth approximately US $30) as an honorarium.

## Results

### Overview

The older people participants in the workshops were aged from 55 to 96 years. An overview of participants for each workshop can be found in Table 1.

**Figure 5:**
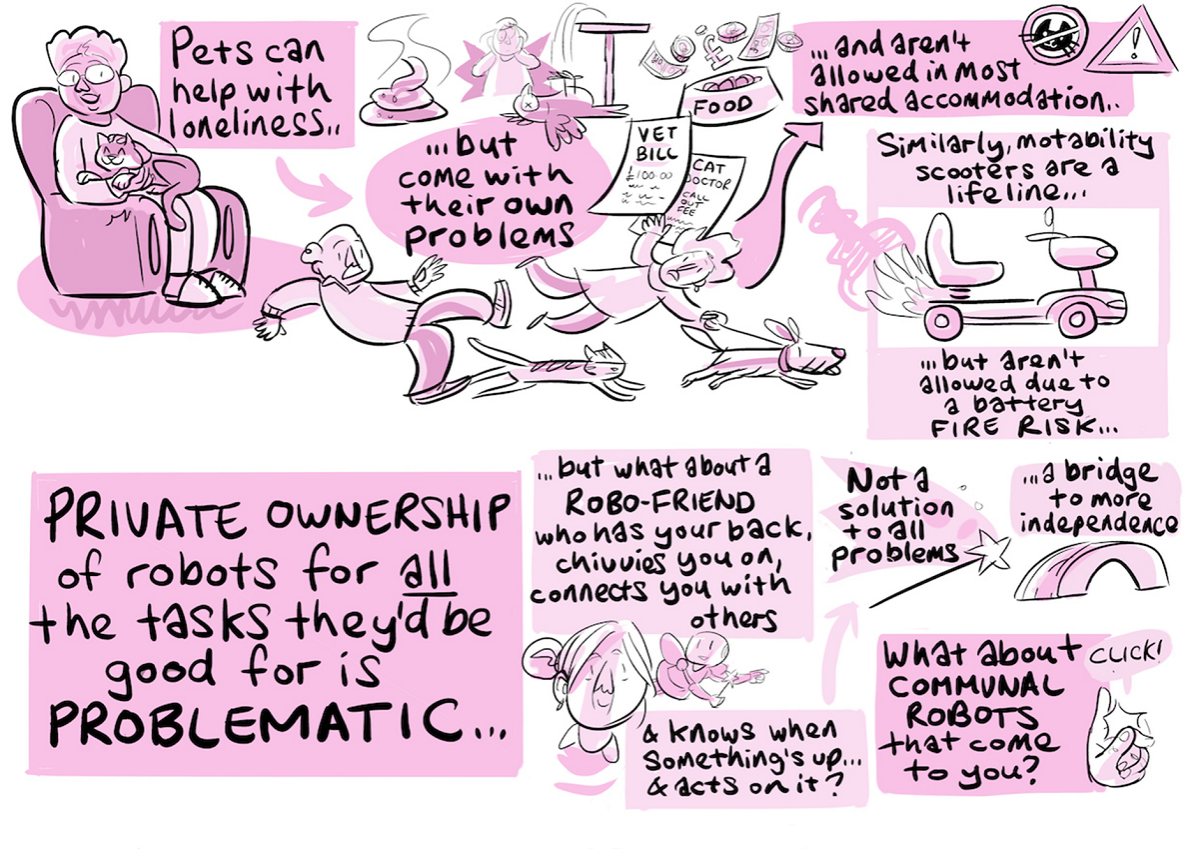
Tableau illustration of discussions during a lived-experience workshop (location A).

**Figure 6:**
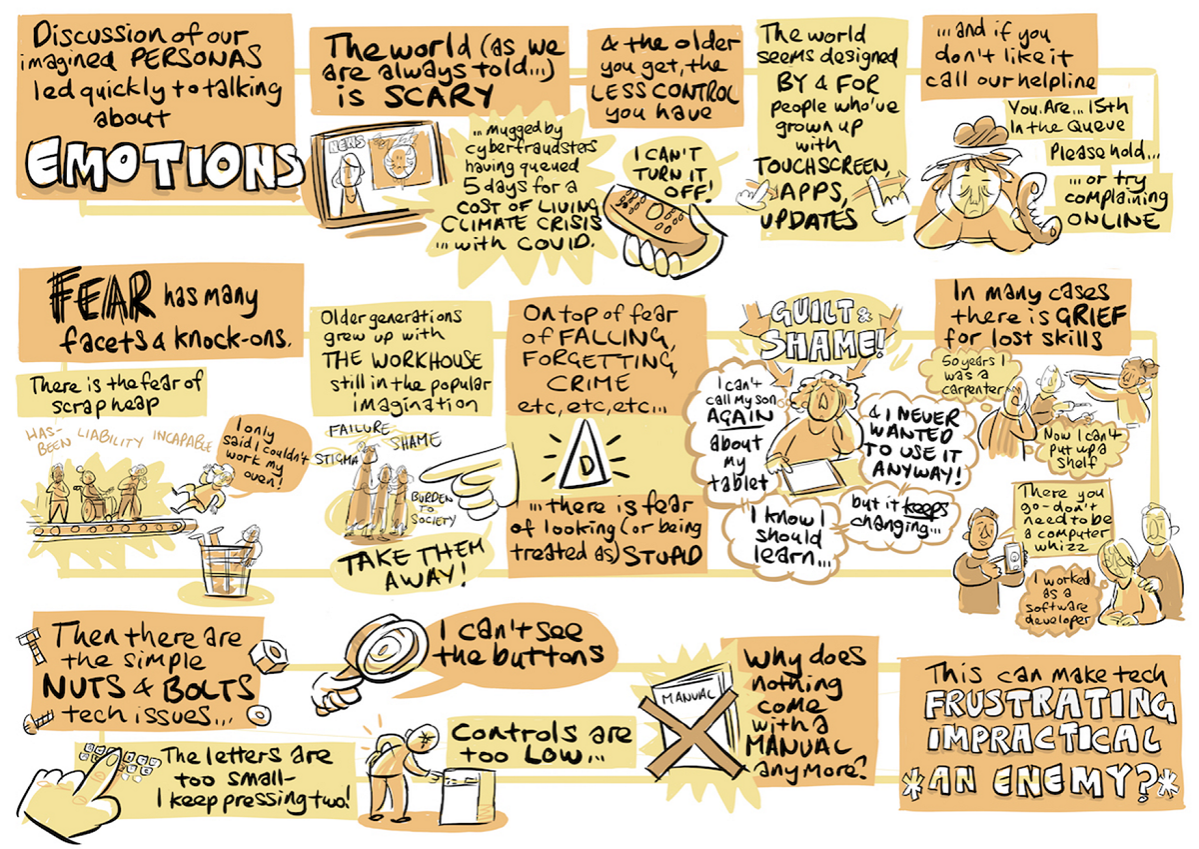
Tableau illustration of discussions during a lived-experience workshop (location C).

The examples of the illustrations produced during the workshops, shown in Figures 5 and 6, give some idea of the content of the discussions.

The analysis of the workshop outputs led to the formation of 3 broad, high-level categories, namely “everyday difficulties,” “ideas for aging better,” and “living with technology.”

**Table 1:** Participant information for each of the co-design workshops.

Workshop type	Location	Participants			Setting
		Older people	Relatives, friends, and caregivers		
L^a^	A	8 (4 F^b^, 4 M^c^)	1 (1 F)	Lounge in residential scheme	
L	B	8 (5 F, 3 M)	5 (3 F, 2 M)	University collaborative space	
L	C	4 (2 F, 2 M)	4 (2 F, 2 M)	University collaborative space	
S^d^	A	5 (3 F, 2 M)	2 (2 F)	Lounge in residential scheme	
S	B	8 (5 F, 3 M)	3 (2 F, 1 M)	University collaborative space	
S	C	4 (2 F, 2 M)	4 (2 F, 2 M)	University collaborative space	

^a^L: lived experience.

^b^F: female.

^c^M: male.

^d^S: speculative critique.

### Everyday Difficulties

The most prevalent codes in the category of everyday difficulties are physical difficulties and psychological difficulties, with other difficulties being less prominent, if not less significant for participants.

- Physical difficulties: as was intended through the format of the lived-experience workshops, physical difficulties were often expressed in terms related to activities of daily living or situated in contexts. For example, participants mentioned the difficulties they have doing the housework (reaching high places, cleaning windows, and making the bed), with personal care (difficulties washing themselves and getting dressed), toileting (incontinence), mobility (walking up hills and stairs, and getting on and off buses), and, in general terms (not exercising enough).
- Psychological difficulties: almost equally prevalent were psychological difficulties. That these are shared by many participants might have been expected; what was more surprising was the extent to which participants were prepared to discuss their own psychological difficulties (and in the process setting aside the fictitious problems faced by the personas). Some of these were related to specific activities; others were more generalized states of mind or mood. Among other things, participants mentioned anxiety (about their own situations, but also more generalized, about the state of the world), social isolation and loneliness, lack of motivation and purpose, depression, and fear (of falling, of going out at night, when answering the door, and of assistance not being available when most needed).
- Cognitive difficulties: principally these concern remembering (appointments, where things are, to take medication, and to recharge and use technology, such as a smartwatch intended to help monitor activity levels).
- Living environment difficulties: these included having limited opportunities to socialize, poor relations with neighbors, lack of convenient public transport, and being unable to keep pets or mobility scooters in shared accommodation (the latter due to size and safety concerns, possibly having implications for assistive robotics).
- Burden of disease: for the workshop participants, this burden included remembering to take medication (overlapping with cognitive difficulties) and maintaining medication routines, the detrimental side effects of medication, and having to care (in an unpaid capacity) for a spouse living with more severe frailty.
- Time pressures: the time required to perform certain activities dissuaded some participants from doing potentially beneficial things such as buying food and preparing meals, taking care of pets, and going out to exercise or socialize.
- Financial difficulties: the cost of living, and specifically the cost of gas and electricity, was mentioned by several participants (who worried that they would be unable to afford to operate robots or other assistive technologies).

### Ideas for Aging Better

Participants also brought to the workshops their own ideas, some based on their own experiences of what works for them, for how people might age better. Some of these concerned living and lifestyle (for instance, exercises, stimulating games, even pet ownership as a motivation for staying engaged and active) and social ideas (conversation and group activities of various sorts). However, the most prevalent category was technological ideas, no doubt influenced by the wider context of the workshops and suggested by the problems faced by the personas (and by the participants themselves); these were further divided into:

- Technology for physical assistance: the category for which there were most suggestions, which included the feasible (and in some cases already available), such as smart speakers for home automation, robot vacuum cleaners, and teasmaids; adaptations of existing technology to the domestic sphere (escalators and self-cleaning toilets, which a participant had seen at an airport); voice-activated showers and personalized drinks and snacks dispensers; and more ambitious ideas involving robots: robots for helping to do the shopping, for cleaning high places, and for cleaning up after pets, automated kitchen surfaces and food packaging openers, and self-making beds.
- Technology for psychological assistance: including technology that tells you who is knocking at the front door, assuring that it is safe to open the door; robots for providing motivation, for recipe suggestions, for cultivating positive attitudes and providing “positive affirmations,” and robotic cats for companionship.
- Technology for cognitive assistance: including devices for providing reminders (for doctors’ appointments or social events, for medicines, food, and drink), and smartwatch navigation aids.
- Technology for social assistance: several participants noted that their smartphones where helping them to stay connected to friends and family, especially through multimodal (speech, video, and chat message) calls; another suggestion was for an app or similar that could allow caregivers to better communicate and coordinate their efforts; and shared smart facilities, such as “robot washing machines” could help to avoid conflicts in residential schemes and similar multihabitant environments.
- Technology for health monitoring: participants suggested that it would be beneficial to have technology that could detect if medication had actually been taken, for measuring nutrients in the body, and for raising the alarm in the case of a fall; and to have an all-purpose “well-being robot.”

Some of the ideas above would find expression in the hypothetical robot designs developed for the speculative critique workshops.

### Living With Technology

The final major category, living with technology, encompasses mainly (but not exclusively) codes identified during analysis of the speculative critique workshops. These cover general preferences (in turn covering aspects such as appropriate interface modalities several people expressed a desire—indeed, an expectation—that the robots would be voice-controlled), size (large enough to do the job, but small enough to fit into people’s homes and lives) and appearance (nonthreatening, with some opting for humanoid and others for “cute” robots), but also ideas of “personalization” (sometimes dynamic or autonomous) of the robot to the user’s (changing) needs, abilities and routines, with robot autonomy extending to regular maintenance tasks such as charging or cleaning itself, and even consideration of financial models for provision (such time-sharing robots with others).

There was some overlap here with general requirements for robots, with a requirement being identified when a participant used a modal verb expressing necessity for some feature or aspect (that a robot “needs” or “must have” it). Requirements refer to physical qualities (robustness and stability), maintenance (cleanable or even self-cleaning), the need for adequate training and practice in using the robot, and, on a more challenging level, the need for a robot to be “self-aware” enough to be able to explain what it is doing.

However, the code with the most items in this category is concerns about technology, which are, almost exclusively, concerns about robotics. These are wide-ranging, revealing the complexity of people’s lives and how they imagine robots might disrupt those. The following subcodes give some flavor of the range of concerns (note that these codes are not mutually exclusive):

- Safety: perhaps unsurprisingly, the major source of concern for the participants is safety. Difficulties here were further coded as surrounding the physical safety of users and other beings in the vicinity of robots, psychological safety (specifically if the appearance of a robot might scare or upset the user or others, manipulate their sentiments, or increase social isolation), potentially detrimental side effects (such as discouraging activity or suggesting the wrong sort of exercise), and digital safety (digital surveillance, privacy, and unauthorized access to any data collected). A number of concerns here are related to what might be termed uncertain hazards, where the participants could envisage situations during the use of the robots in which their safety could be imperiled if appropriate safeguards are not in place. For instance, what happens if a robot intended to physically assist people up and down stairs, or a flying drone-type cleaning robot were to malfunction midoperation?
- Compatibility: participants raised practical concerns about how robots would fit into their everyday lives, including aspects such as social compatibility (for instance, will the neighbors be inconvenienced by a robot? How would it interact with pets?), physical compatibility (Where will the robot be stored? Will it require modifications to the home? Will it damage possessions?), and technical compatibility (Will it require broadband connectivity?).
- Usefulness-desirability-inclusivity: general questions were raised about the usefulness of the speculative robots, including whether there were simpler or cheaper ways of achieving the same ends using existing technology (such as tablet computers and smart speakers, and nondigital alternatives), about the longevity of the robots (would they end up gathering dust in the cupboard alongside the foot spa?), about the need for sensitive, nonstigmatizing, design of robots (and other assistive technologies), and, indeed, whether there was any real need for robots at all (“Is this a generational thing?” asked a participant; and another: “Am I going to feel the same about robots as I do about other tech? Too much hassle!”)
- Burden of use: the additional overheads of looking after a robot are another major source of concern. These overheads encompass cleaning, charging, and servicing robots, the training required, and the cognitive demands of remembering how to use the robot. To quote a participant, “We don’t want to look after the robot–we want the robot to look after us!”
- Maintenance and service model: a related concern is the service model of which the robot constitutes a technical component (and which is often overlooked in robot development): who programs, sets up, and personalizes the robot? Do robots act alone or in concert with human assistants? Who is responsible for regular maintenance and repairing faults?
- Control: concerns were raised about how control is exercised over robots, and who is in control. Some of these relate to the immediate operation of the robot, especially where the behaviors are complicated or potentially hazardous (and, in the words of a participant, “Can you tell it to stop?”), and others to the underlying operation of the robot, such as who determines what type and amount of motivation or exercise a robot should suggest.
- Financial costs: the economics of assistive robotics were also raised: participants raised the costs of purchase, running, and maintaining a robot, but also of modifying their homes, if necessary. Some participants asked about the comparative costs, whether that be compared with a human caregiver providing the same services or with a conventional refurbishment of their homes to make them more “age-friendly.”
- Techno-psychosocial effects: here we see what might be called the techno-psychosocial effects of aging. These include the fear of being deemed “obsolete” or a burden on society, in no small part due to a (real or perceived) inability to move with the times and adapt to new technologies, as well as more “conventional” fears of falling, or forgetting, or crime and so on; the guilt and shame of not being able to adapt to these new technologies (alongside a feeling of grief for those skills that by-and-by are lost as we age); the stigma that sometimes accompanies the use of assistive technologies; the bottled-up frustration and occasional releases of rage that come from dealing with an increasingly digital world; and the contribution all these factors make to a vicious circle of stress, anxiety, and depression, with accompanying relationship problems, lack of motivation, poor diet, and social isolation.
- Digital ethics issues: in addition to the ethical issues that run through many of the difficulties noted above, here we also encounter direct concerns around the surveillance that users of data-collecting robots might find themselves subject to, users’ privacy, and the security of their data.
- Social and care implications: finally, several participants raised concerns about the wider impact of assistive robotics: its potential negative effect on the role and jobs of human caregivers and home-helps, and the worry that robot care might replace human care, with the assumption that a degradation in the quality of that care would necessarily follow. On the other hand, there are worries too about inclusivity, access to care services, and the place of those who are unable to adapt to—or who choose to opt out of—the brave, new digital world.

## Discussion

### Principal Findings

We aimed to gain a better understanding of the everyday lives of older people, especially with reference to the effects of aging and frailty, and to gauge their opinions and concerns about assistive robotics. Through a wide-ranging co-design exercise with older people and their caregivers, we have gained a compelling snapshot of older people’s real lives. As might be expected, given the structure of the workshops, the emphasis lies on the problems that people face daily and on their concerns about a world where change seems to have accelerated, sweeping away old certainties, and where, increasingly, services are delivered using digital technologies about which they have little say and less control [42]. In this context, the work reported here is an attempt to redress this balance and to return to older people some agency in the development of assistive technologies. We have also highlighted general concerns that older people have about assistive robotics. While older people and their caregivers are open to, and in some cases, enthusiastic about, the role that assistive robotics could play in their lives and those of others, they raise real concerns about, among other things, safety and control, the burden of use, ethics and unintended side effects, and the financial and social costs of introducing robots into their lives.

The work reported here differs from previous assistive robotics co-design activities in several key aspects. First, in its scope, which embraced a wide range of experiences of growing older, with a focus on the individual rather than on some specific health condition or difficulty, as much previous work has tended to do (eg, helping mitigate dementia [43] or supporting mobility [22,26]). Second, the exercise was technology-neutral in that we did not structure the workshops around some particular robot or robotic platform and tried to stay impartial in the question of whether assistive robotics could be beneficial: to adopt a priori a particular robotic platform is to make design decisions, including the fundamental decision that a robot is a valid “solution” to an ill-defined problem [27]. Finally, the exercise was undertaken not as a step in a specific assistive-robotics development pathway, but as a service to the wider assistive robotics community exploiting the Emergence network’s resources, skills, and access to engage with potential users, and as such, we have placed special emphasis on communicating the results. In methodological terms, rather than focusing on a particular problem or solution (as is often done in co-design activities), we chose to adopt a general approach and investigate the lives of older people more broadly, and without trying to reach any firm consensus about where to focus subsequent development efforts or trying to reconcile the at times contradictory opinions of participants. We hope that, by explaining our co-design approach along with the results, assistive technology developers will be able to sift the evidence and draw sound, rational conclusions.

### Communication and Dissemination of Results

**Figure 7:**
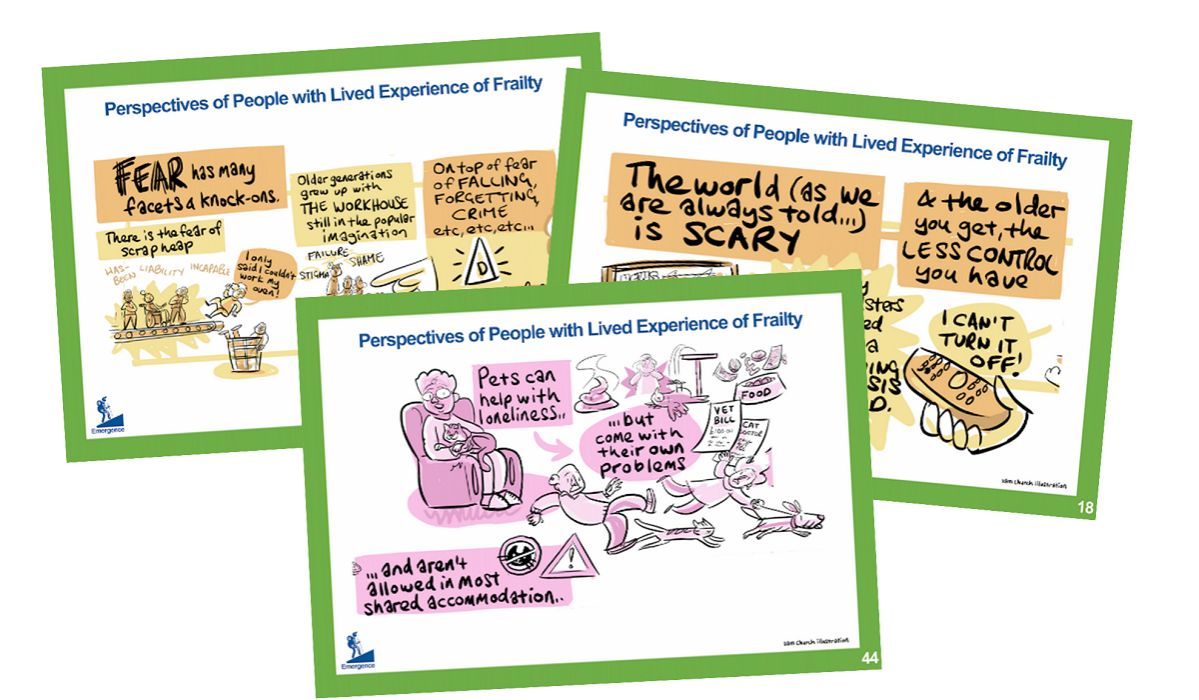
Some of the empathy cards generated from tableaux vignettes.

One key aspect of the exercise reported here is the communication and dissemination of results: clearly, this is essential if the findings are to influence the development of assistive robotics. One novel aspect here is that we realized that the results of the graphic illustrator’s work to document the workshops could come to play an innovative role in our dissemination activities. The illustrator produced a total of 11 tableaux from the 3 lived experience workshops (Figures 5 and 6 each show 1 of these tableaux). Each tableau, somewhat like a comic book page, consists of a number of thematically related vignettes illustrating the ideas, opinions, or concerns voiced by participants. It was realized that these vignettes could form powerful communication and design aids as self-contained glimpses into the lives of the participants. Accordingly, they were isolated and printed as a pack of playing card-sized “empathy cards” (Figure 7), complete with suggestions for workshop “games” that encourage players to explore the different perspectives, concerns, and opportunities the cards contain and to consider how these relate to their own projects.

Along with the empathy cards, the results of the analysis have formed the basis of a series of workshops, sandpits, and project funding calls, and have provided content for a multidisciplinary summer school for budding assistive robotics developers. Through these activities, we have tried to reorient the development of assistive robotics for older people in a more empathetic direction and, indeed, to foment a new generation of more empathetic roboticists.

In addition, we are in the process of making all our workshop materials and the analysis results open and accessible in digital formats to the wider robotics and assistive technologies communities. This content includes the personas, the speculative robotics designs, the results of the analysis, and the empathy cards (which are also available as a physical pack of cards). The process and content of communicating outcomes in an accessible, understandable, and actionable manner is something that is not always given due prominence when discussing co-design exercises. This paper constitutes an element of our communication strategy, but it is by no means the culmination of our labors.

At the time of writing, it is too soon to draw any firm conclusions about the success of these endeavors: the development of assistive technologies and, in particular, of assistive robotics is a difficult and slow process which, typically, extends over several years. Only time will tell whether our efforts have contributed to bringing about the intended effect, and we see assistive robots emerge from the laboratory to help older people in their everyday lives.

### Limitations

We recognize a number of limitations of this co-design exercise. All co-design practice is subject to bias, and this is no exception. The structure of the workshops, the development and choice of the material that provided the stimuli for them, the data collection by facilitators during the workshops, and the analysis of that data are all prey to the biases and preconceived notions of the researchers. The co-design approach adopted was, in some sense, an attempt to counter these biases: by not focusing on any particular everyday problem or given robotic platform, we have attempted to give participants the freedom to steer the discussions. In addition, the multidisciplinary nature of the research team (which included specialists in design, assistive technologies, and health and care provision, as well as robotics) helped to guard against adopting preconceived positions or attitudes.

Further limitations concern the representativeness of the participants. The co-design activities involved a total of only 20 older people, plus a smaller number of relatives, friends, and caregivers. The participants were healthy enough (or coping well enough) to attend workshops and possessed some degree of day-to-day independence. Hence, people living with more severe frailty or other age-related health conditions (such as dementia) were not represented directly in the workshops, and no consideration was given to the particularities of life in care homes and other facilities offering specialized care (although the use of personas with more severe frailty and health issues was an attempt to address, at least in part, these aspects). Clearly, there is much scope for developing assistive robotics for people with greater needs and for the staff who minister to those needs, but that would require other, more focused co-design exercises. To this end, we have also conducted workshops with health care professionals, which—as may be expected—focused more specifically on the needs of people with more severe frailty and health conditions (including end-of-life care) and with a greater emphasis on support for their caregivers. As the outcomes of these workshops are thematically quite different from those reported here, this work will be reported separately.

Furthermore, the participants in this exercise were self-selecting, and as such might be considered to have a greater interest in robotics (and digital technologies more generally), and possibly to be better disposed to the acceptance of domestic assistive robotics. We did not collect data about the socioeconomic status or the digital literacy and experience of participants, factors that can influence attitudes to robotics. It was not feasible to reconvene participants to confirm or correct the analysis of the workshop data.

In demographic terms, all the older participants self-identified as having a White British ethnic background (almost certainly a reflection of the constitution of independent-living schemes from which most participants were recruited rather than any explicit bias in recruitment practices). Looking beyond to other, perhaps underserved, communities and their living circumstances could expose a different set of needs and wants. Although we found little direct evidence of differences in attitudes according to age or gender, these moderators will likely have some influence on acceptance, even if it was not explored by our participants. To assume that all older people will have the same attitudes to technology, disregarding other factors in their background, is to risk adhering to ageist stereotypes [44]. The very term “older people,” with the suggestion it conjures of a homogeneous mass of people, can itself be a barrier. However, we would argue that experience is a more influential moderator than age: many older people, including some of our workshop participants, will have had extensive experience with digital technology throughout their working lives (which, in the case of a couple of participants, was ongoing) or because they have readily embraced it in their social lives, and are receptive to and comfortable with the idea of using digital assistive devices. Studies have suggested that perceived value and benefit of new technologies are more significant for technology acceptance than is chronological age [45,46]. Rather than being technophobic as a rule or having low levels of digital literacy, older people seem less likely to invest time in new digital technologies whose value for them is not apparent. However, while we have striven throughout to avoid adopting ageist stereotypes, we are aware that this is an all-too-easy pitfall (and, indeed, we noted during the workshops that older people themselves sometimes fall back on these facile generalizations).

In summary, we could not hope to capture everyone’s experience of frailty and aging, or even to encompass the complexity of a single individual’s experience. Moreover, the results of this exercise are specific to a particular population, time, and place (and as such have a limited shelf-life): namely, they are valid for (a small subset of) older people living in the United Kingdom in the early 2020s. People’s experiences in different places and times will, of course, be different. If we were to repeat this exercise in, say, a generation’s time, we would expect the needs and expectations of older people to diverge dramatically from those seen in our results. By this measure, there is no such thing as a uniform, constant, consistent experience of frailty and aging. In some sense, this is not a failing of this exercise but rather an essential feature of co-design, albeit one rarely acknowledged in the literature. Design always responds to problems and opportunities situated in particular times and places; seen in this light, this is not a limitation of the snapshot, but a fundamental aspect of it. Good designs, or ones that address a particular need, might be able to transcend time and place to some extent, but of necessity they must be grounded in the here-and-now experience of everyday life.

### Conclusions

The contributions of this paper can be summarized as follows. We have described the predominant clinical models of frailty and explained why these form a useful but incomplete basis for designing assistive technologies of any sort for people living with frailty and older people more generally. We have described the current underwhelming state of assistive robotics, with common failings during their design or co-design processes that often result in the development of inappropriate robotic services.

We have described a novel co-design methodology that has attempted to combine persona-based lived-experience workshops with provotype-based speculative-design workshops with older people to gain a rounded “phenomenological snapshot” of the experience of being old in an increasingly digital world, a world in which assistive robotics may soon become commonplace. As far as we are aware, this is the first time that an exercise of this scope has been attempted in the context of assistive robotics. Finally, we have outlined the major concepts that are revealed by an analysis of the results of the workshops. These can be categorized thematically as: everyday difficulties, the problems faced daily by people living with frailty and older people more generally; ideas for aging better, older people’s own suggestions for how their lives could be improved; and living with technology. In addition to participants’ preferences and requirements, this last category reveals the wide-ranging concerns that people have about services based on digital technologies, and about assistive robots in particular, whose development is viewed with a mixture of enthusiasm and unease.

## Data Availability

The data generated and analyzed during this study are available from the corresponding author (SP) on reasonable request.

## References

[1] Wright J (2003). Robots Won't Save Japan: An Ethnography of Eldercare Automation.

[2] Xue Q (2011). The frailty syndrome: definition and natural history. Clin Geriatr Med.

[3] Clegg A, Bates C, Young J, Ryan R, Nichols L, Ann Teale E, Mohammed MA, Parry J, Marshall T (2016). Development and validation of an electronic frailty index using routine primary care electronic health record data. Age Ageing.

[4] Fried LP, Tangen CM, Walston J, Newman AB, Hirsch C, Gottdiener J, Seeman T, Tracy R, Kop WJ, Burke G, McBurnie MA, Cardiovascular Health Study Collaborative Research Group (2001). Frailty in older adults: evidence for a phenotype. J Gerontol A Biol Sci Med Sci.

[5] Walston JD, Bandeen-Roche K (2015). Frailty: a tale of two concepts. BMC Med.

[6] Rockwood K, Andrew M, Mitnitski A (2007). A comparison of two approaches to measuring frailty in elderly people. J Gerontol A Biol Sci Med Sci.

[7] Rockwood K, Mitnitski A (2007). Frailty in relation to the accumulation of deficits. J Gerontol A Biol Sci Med Sci.

[8] Lang IA, Llewellyn DJ, Langa KM, Wallace RB, Huppert FA, Melzer D (2008). Neighborhood deprivation, individual socioeconomic status, and cognitive function in older people: analyses from the English Longitudinal Study of Ageing. J Am Geriatr Soc.

[9] Werner C, Moustris GP, Tzafestas CS, Hauer K (2018). User-oriented evaluation of a robotic rollator that provides navigation assistance in frail older adults with and without cognitive impairment. Gerontology.

[10] Koumpouros Y, Toulias TL, Tzafestas CS, Moustris GP (2020). Assessment of an intelligent robotic rollator implementing navigation assistance in frail seniors. Technol Disability.

[11] Ozaki K, Kondo I, Hirano S, Kagaya H, Saitoh E, Osawa A, Fujinori Y (2017). Training with a balance exercise assist robot is more effective than conventional training for frail older adults. Geriatr Gerontol Int.

[12] Hai NDX, Thinh NT (2022). Self-feeding robot for elder people and Parkinson‘s patients in meal supporting. Int J Mech Eng Robot Res.

[13] Luperto M, Monroy J, Renoux J, Lunardini F, Basilico N, Bulgheroni M, Cangelosi A, Cesari M, Cid M, Ianes A, Gonzalez-Jimenez J, Kounoudes A, Mari D, Prisacariu V, Savanovic A, Ferrante S, Borghese NA (2023). Integrating social assistive robots, IoT, virtual communities and smart objects to assist at-home independently living elders: the movecare project. Int J Soc Robot.

[14] Pollak C, Wexler SS, Drury L (2022). Effect of a robotic pet on social and physical frailty in community-dwelling older adults: a randomized controlled trial. Res Gerontol Nurs.

[15] Yamazaki Y, Ishii M, Ito T, Hashimoto T (2021). Frailty care robot for elderly and its application for physical and psychological support. J Adv Comput Intell Intell Inform.

[16] Boumans R, van Meulen F, Hindriks K, Neerincx M, Olde Rikkert MGM (2019). Robot for health data acquisition among older adults: a pilot randomised controlled cross-over trial. BMJ Qual Saf.

[17] Civit A, Andriella A, Barrue C, Antonio M, Boqué C, Alenyà G (2024). Introducing social robots to assess frailty in older adults.

[18] Olde Keizer RACM, van Velsen L, Moncharmont M, Riche B, Ammour N, Del Signore S, Zia G, Hermens H, N’Dja A (2019). Using socially assistive robots for monitoring and preventing frailty among older adults: a study on usability and user experience challenges. Health Technol.

[19] Arunachalam S (2023). Do socially assistive robots help in caring for elderly patients with frailty or mild cognitive impairment?. J Stud Res.

[20] Lunardini F, Luperto M, Romeo M, Renoux J, Basilico N, Krpic A, Borghese N, Ferrante S (2019). The MOVECARE project: home-based monitoring of frailty.

[21] Kim J, Choi Y, Jeong S, Han J (2022). A care robot with ethical sensing system for older adults at home. Sensors (Basel).

[22] Fiorini L, Tabeau K, D’Onofrio G, Coviello L, De Mul M, Sancarlo D, Fabbricotti I, Cavallo F (2020). Co-creation of an assistive robot for independent living: lessons learned on robot design. Int J Interact Des Manuf.

[23] Steen M (2013). Co-design as a process of joint inquiry and imagination. Design Issues.

[24] García-Soler Á, Facal D, Díaz-Orueta U, Pigini L, Blasi L, Qiu R (2018). Inclusion of service robots in the daily lives of frail older users: a step-by-step definition procedure on users' requirements. Arch Gerontol Geriatr.

[25] Fiorini L, D'Onofrio G, Rovini E, Sorrentino A, Coviello L, Limosani R, Sancarlo D, Cavallo F (2019). A robot-mediated assessment of Tinetti balance scale for sarcopenia evaluation in frail elderly.

[26] Coviello L, Cavallo F, Limosani R, Rovini E, Fiorini L (2019). Machine learning based physical human-robot interaction for walking support of frail people.

[27] Bardaro G, Antonini A, Motta E (2022). Robots for elderly care in the home: a landscape analysis and co-design toolkit. Int J Soc Robotics.

[28] Spiers J, Smith JA, Atkinson P, Delamont S, Cernat A, Sakshaug JW, Williams RA (2019). Interpretative phenomenological analysis. SAGE Research Methods Foundations.

[29] Pruitt J, Adlin T (2006). The Persona Lifecycle: Keeping People in Mind Throughout Product Design.

[30] van den Heuvel H, Huijnen C, Caleb-Solly P, Nap H, Nani M, Lucet E (2012). Mobiserv: a service robot and intelligent home environment for the provision of health, nutrition and safety services to older adults. Gerontechnology.

[31] Wöckl B, Yildizoglu U, Buber I, Aparicio Diaz B, Kruijff E, Tscheligi M (2012). Basic senior personas: a representative design tool covering the spectrum of European older adults.

[32] Valenza C, Adkins J (2009). TIMELINES: understanding visual thinking: the history and future of graphic facilitation. Interactions.

[33] Espiner D, Hartnett F (2016). Innovation and graphic facilitation. Aotearoa New Zealand Social Work.

[34] Boer L, Donovan J (2012). Provotypes for participatory innovation.

[35] Dunne A, Raby F (2013). Speculative Everything: Design, Fiction, and Social Dreaming.

[36] Sellen KM, Massimi MA, Lottridge DM, Truong KN, Bittle SA (2009). The people-prototype problem: understanding the interaction between prototype format and user group.

[37] Hogan DB, MacKnight C, Bergman H, Steering Committee‚ Canadian Initiative on Frailty and Aging (2003). Models, definitions, and criteria of frailty. Aging Clin Exp Res.

[38] Ritchie J, Lewis J (2003). Qualitative Research Practice: A Guide for Social Science Students and Researchers.

[39] Gale NK, Heath G, Cameron E, Rashid S, Redwood S (2013). Using the framework method for the analysis of qualitative data in multi-disciplinary health research. BMC Med Res Methodol.

[40] Tuffour I (2017). A critical overview of interpretative phenomenological analysis: a contemporary qualitative research approach. J Healthc Commun.

[41] Neubauer BE, Witkop CT, Varpio L (2019). How phenomenology can help us learn from the experiences of others. Perspect Med Educ.

[42] (2024). Briefing: facts and figures about digital inclusion and older people. Age UK.

[43] Moharana S, Panduro AE, Lee HR, Riek LD (2019). Robots for joy, robots for sorrow: community based robot design for dementia caregivers.

[44] Mannheim I, Wouters EJM, Köttl H, van Boekel LC, Brankaert R, van Zaalen Y (2023). Ageism in the discourse and practice of designing digital technology for older persons: a scoping review. Gerontologist.

[45] Hauk N, Hüffmeier J, Krumm S (2018). Ready to be a silver surfer? A meta-analysis on the relationship between chronological age and technology acceptance. Comput Hum Behav.

[46] Berkowsky RW, Sharit J, Czaja SJ (2018). Factors predicting decisions about technology adoption among older adults. Innov Aging.

